# Disinfection of various materials with 3-(trimethoxysilyl)-propyldimethyloctadecyl ammonium chloride in hatchery facilities

**DOI:** 10.5713/ab.21.0302

**Published:** 2021-10-21

**Authors:** Yu-Jin Kim, Jun-Beom Kim, Chang-Seon Song, Sang-Soep Nahm

**Affiliations:** 1 College of Veterinary Medicine, Konkuk University, Seoul 05029, Korea; 2 KCAV Co., Ltd., Seoul 05029, Korea

**Keywords:** Biosecurity, Disinfection, Field-Emission Scanning Electron Microscopy (FE-SEM), Hatchery, Long-lasting Disinfectant, Si-QAC

## Abstract

**Objective:**

Surface disinfection is important in the proper running of livestock farms. However, disinfection of farm equipment and facilities is difficult because they are made of different materials, besides having large surface areas and complex structures. 3-(trimethoxysilyl)-propyldimethyloctadecyl ammonium chloride (Si-QAC) is a quaternary ammonium salt-based disinfectant that attaches to various surfaces by forming covalent bonds and maintains its disinfecting capacity for a considerable time. Our aim was to evaluate the potential use of Si-QAC for disinfection of farm equipment and facilities.

**Methods:**

The short- and long-term antimicrobial and antiviral effects of Si-QAC were evaluated in both laboratory and farm settings using modified quantitative assessment method based on the standard operating procedures of the United States Environmental Protection Agency.

**Results:**

Si-QAC was highly effective in controlling the growth of the Newcastle disease virus and avian pathogenic *Escherichia coli*. Electron microscopy revealed that the mechanism underlying the disinfection activity of Si-QAC was associated with its ability to damage the outer membrane of the pathogen cells. In the field test, Si-QAC effectively reduced viral contamination of surfaces of equipment and space.

**Conclusion:**

Our results suggest that Si-QAC has great potential as an effective chemical for disinfecting farm equipment and facilities. This disinfectant could retain its disinfection ability longer than other commercial disinfectants and contribute to better farm biosecurity.

## INTRODUCTION

According to the report of the World Organization for Animal Health (OIE) World Animal Health Information System (WAHIS), more than 6,000 Highly Pathogenic Avian Influenza (HPAI) cases have occurred during 2016 through 2020, resulting in the disposal of more than 100 million poultries [1]. The transmission of HPAI from one farm to another is believed to be the main cause of massive outbreaks. The HPAI epidemiological investigation report of 2017 through 2018 by the Korean Animal and Plant Quarantine Agency showed that nearly 20% of all HPAI outbreaks were associated with employees working with HPAI-contaminated equipment or vehicles [2]. Similar results were found in the analysis of the 2016 through 2017 HPAI outbreak in France. Researchers in the study found that loosely defined boundaries, which is highly associated with inadequate control of movements and cleaning of facilities, has a decisive effect on HPAI spread in farms [3]. Thus, disinfection becomes essential for the proper protection of animals in livestock farms.

Selection of an appropriate disinfectant from the various commercially available ones depends upon the surface to be disinfected, target pathogen(s), and ambient environment. One challenge faced during surface disinfection is the surface structure, particularly equipment or large machines consist of multi-materials and subtle smaller parts that were too difficult to maintain clean status with a short routine cleaning. Moreover, it is difficult to maintain the disinfected status of a surface for a prolonged period because traditional disinfectants’ efficacy quickly decreases. To overcome this, coating substances with copper or silver, which have sterilizing properties, are often used [4,5]. Materials such as antibacterial glasses or ion creams have been commercially developed to prevent bacterial adhesion and induce bacterial cell death. However, these materials exert disin-fecting effects via leaching of the active components of their chemical structures; thus, there may be a gradual decrease in their disinfecting efficacy upon the contamination of surrounding environments because of a loss of these active components [6]. Importantly, a disinfectant used for the surface disinfection of equipment in livestock facilities should exert a long-lasting effect.

The organosilicon quaternary ammonium salt 3-(trimethoxysilyl)-propyldimethyloctadecyl ammonium chloride (Si-QAC) has been recently developed as a surface disinfectant. Si-QAC has two distinctive parts—organosilane and a quaternary ammonium compound [7]. The organosilicon moiety attaches to various types of surfaces, including steel, plastic, and glass, and it does not leach when completely dried [8]. Quaternary ammonium compounds are cationic surfactants that vary in structure based on the length of their carbon chains. These compounds are widely used as potent disinfectants against a broad spectrum of microorganisms [9]. Because Si-QAC has moieties allowing it to adhere to surfaces and exert disinfecting effects [10]. Several attempts were made to exploit the antimicrobial characteristics of Si-QAC. At the early stage of Si-QAC development, it was applied to fabrics to test antimicrobial effects [11,12]. Recently, experimental results of the use of Si-QAC as a disinfectant on medical implants and dental materials were reported [13,14]. However, the effects of Si-QAC have not been tested on a wide range of facilities such as public facilities or animal farms that require large-capacity, rapid disinfecting process of broad surfaces with high levels of hygiene. Based on the results of previous studies, we anticipated that Si-QAC can be used as a long-term surface disinfectant. Thus, in this study, we evaluated the potential of Si-QAC as a surface disinfectant in both laboratory and farm settings.

## MATERIALS AND METHODS

### Materials

All tested surfaces (stainless steel, polypropylene, and glass) were fashioned into circular pieces (diameter, 1 cm; thickness, 1 to 2 mm) and autoclaved. The Newcastle disease virus (NDV) was obtained from KBNP Inc. (Yesan, Korea), and the avian pathogenic *Escherichia coli* (APEC) was provided by the Avian Disease Laboratory of Konkuk University (Seoul, Korea).

### Experimental design

Disinfection efficacy was assessed using a modified quantitative assessment method based on the standard operating procedures of the United States Environmental Protection Agency [15]. Briefly, 500 μL of Si-QAC (SD Lab, Seoul, Korea) was evenly applied to the test surface. Surfaces that were not treated with Si-QAC were used as controls. The test surfaces were then artificially contaminated with either NDV (10^6^ 50% egg infectious dose [EID50]/mL; 100 μL) or APEC (10^8.5^ colony-forming units [CFUs]/mL; 10 μL) using a micropipette by placing drops of the microbial culture suspensions onto the surface. To determine the minimum time required for antimicrobial and antiviral effects, disinfection efficacy was tested at 10, 20, and 30 min after contamination of uncoated/Si-QAC-coated surfaces. Antimicrobial and antiviral effects were measured by culturing the disinfectant-treated pathogens from the test materials in nutrient agar or in 10-day-old embryonated eggs (Duggi Farm, Yongin, Korea). For long-term disinfection efficacy tests, the same procedure was followed, and the evaluation was performed every 10 days for 30 days.

### Electron microscopy observations

The morphology of disinfectant-treated pathogens was evaluated using field-emission scanning electron microscopy (FE-SEM). After 30 min of incubation on the test surfaces, the pathogens were collected and washed twice with phosphate-buffered saline (PBS). The samples were fixed in two stages—first in Karnovsky’s fixative for 6 h and then in 1% osmium tetroxide dissolved in 0.1 M PBS for 1.5 h. The fixed samples were dehydrated using 10% ascending gradual ethanol solu-tions starting from 60%, followed by treatment with isoamyl acetate. The samples were finally passed through a critical point dryer (EM CPD300; Leica Microsystems Inc., Vienna, Austria). Then, the samples were coated with platinum powder using an ion coater (EM ACE600; Leica Microsystems Inc., Austria) and observed using a field-emission scanning electron microscope (Carl ZEISS, Merin, Germany) at a voltage of 10 kV.

### Field test

Field tests at a hatchery (Gunsan, Korea) were conducted in cooperation with an integrated poultry company in Korea. The equipment and facility surfaces were made of either plastic or stainless steel. After cleaning the surfaces using hot water and detergent, Si-QAC was applied onto the equipment, including the egg trays and chick-hatching trays, and an egg incubation facility. Hundred microliters of NDV were then applied to the equipment and facility surfaces. After 30 min, the viruses were retrieved using sterilized cotton swabs and immersed in PBS; the residual viral titers in these suspensions were quantified by culturing the suspensions in 10-day-old embryonated eggs.

### Statistical analysis

All data were analyzed using the SPSS (version 23) software for Windows (IBM, Armonk, NY, USA). Analysis of variance and Student’s t-test were used to determine the differences between groups. Statistical significance was set at p<0.05.

## RESULTS

### Minimum reaction time of Si-QAC

**Figure 1:**
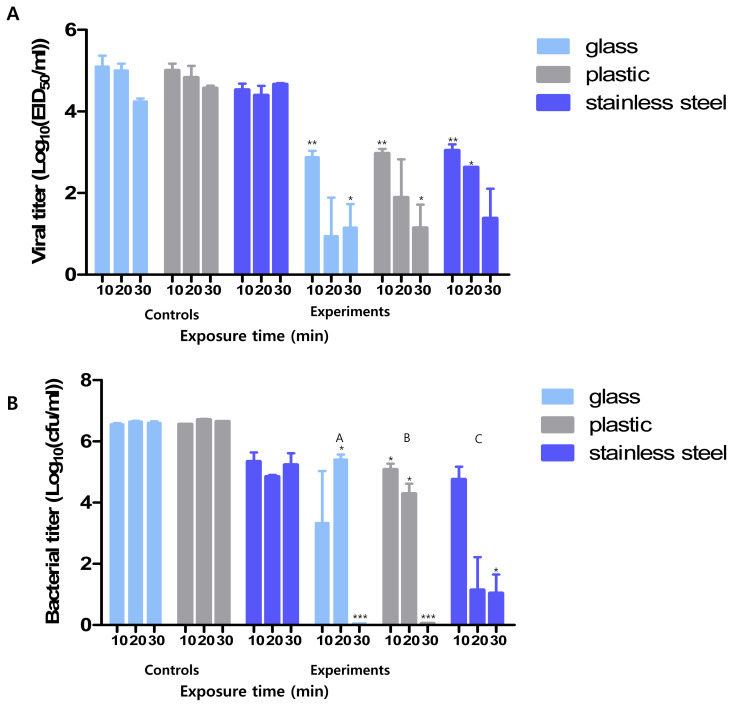
Minimum reaction time of Si-QAC. Minimum time required for the antiviral (A) and antimicrobial (B) effects of Si-QAC on different test surfaces. Si-QAC, 3-(trimethoxysilyl)-propyldimethyloctadecyl ammonium chloride. * p<0.05, ** p<0.005, *** p<0.001 compared to that of the control group at different time intervals. The different letters denote statistical significance over time within a group (p<0.05).

We measured the minimum reaction time required for Si-QAC to exert its disinfection effects. Compared with the control groups, the counts of residual pathogens on the surfaces of most of the tested materials, except glass and stainless steel, in the experimental groups decreased from the 10-min time point. The viral and bacterial titers in all the tested surfaces from the experimental group were notably reduced at 30 min compared to those in all the tested surfaces of the control group (Figure 1).

This disinfectant showed an antiviral effect starting from 10 min compared to the control group. After 30 min, the viral titer for all test surfaces in the experimental group was less than 10^2^ EID50/mL, which was significantly lower than in the control group. Further, the antimicrobial titer in the experimental group after 30 min was less than 10 CFUs/mL, which was significantly lower than in the control group (10^6^ CFUs/mL).

### Long-term disinfection efficacy

**Figure 2:**
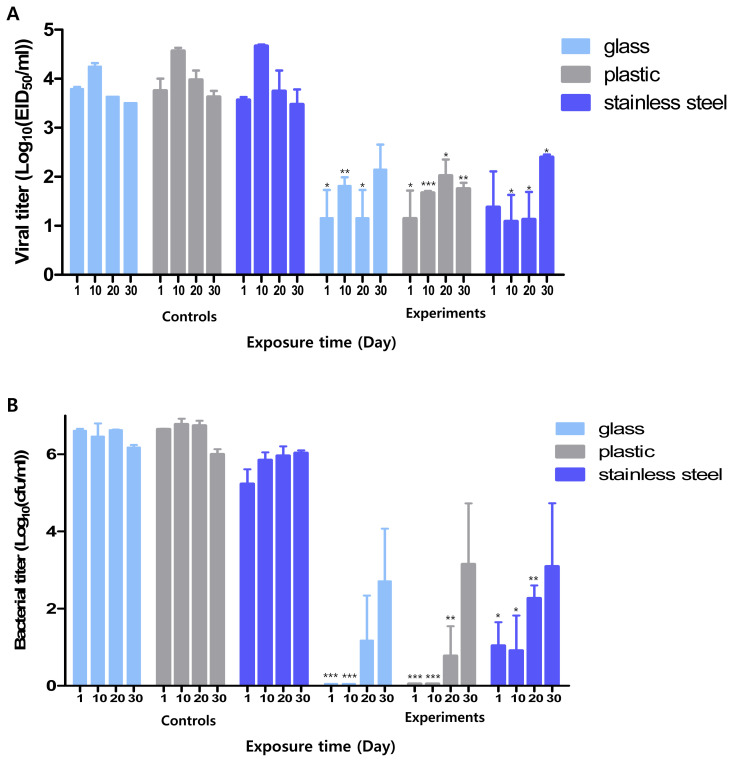
Long-term disinfection efficacy of Si-QAC: Long-term antiviral (A) and antimicrobial (B) effects of Si-QAC on different surfaces. Si-QAC, 3-(trimethoxysilyl)-propyldimethyloctadecyl ammonium chloride. * p<0.05, ** p<0.005, *** p<0.001 compared to that in the control group at different time intervals.

To evaluate the duration of the disinfection effect of Si-QAC on the test surfaces, its antiviral and antimicrobial effects were monitored over a month (Figure 2). In the experimental group, antiviral and antimicrobial effects were maintained for at least 20 days on all the tested surfaces (p<0.05). Notably, the antiviral effect was maintained for 20 days on glass and 30 days on plastic and stainless steel. Within the experimental group, no statistical differences in viral titers were observed across the time points for the test surfaces. Evaluation of the long-term antimicrobial effects of Si-QAC yielded comparable results. The bacterial titers on all test surfaces in the experimental group were significantly lower than those on all test surfaces in the control group for up to 20 days. Within the experimental group, no statistical differences in bacterial titers were observed across the time points for the test surfaces.

### Field-emission scanning electron microscopy observations

**Figure 3:**
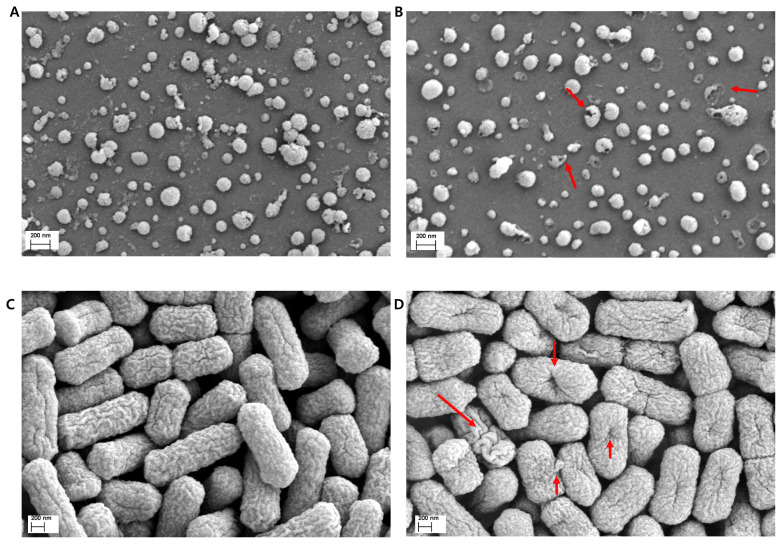
FE-SEM analysis of pathogens revived from test surfaces treated with Si-QAC: FE-SEM images showing the morphology of normal pathogens (A, NDV; C, APEC) and disinfectant-treated pathogens (B, NDV; D, APEC). The red arrows indicate severe damage. FE-SEM, field-emission scanning electron microscopy; Si-QAC, 3-(trimethoxysilyl)-propyldimethyloctadecyl ammonium chloride; NDV, Newcastle disease virus; APEC, avian pathogenic *Escherichia coli*.

Field-emission scanning electron microscopy analysis revealed that the outer membrane of the pathogen cells was damaged after treatment with Si-QAC (Figure 3). NDV particles with damaged or perforated envelopes were observed more frequently in the experimental group than in the control group (Figure 3A, B). Most of the bacterial cells appeared to be relatively smooth and intact in the control group. However, images obtained from the test surfaces in the experimental group showed many indentations and cracks on the bacterial wall and shrunken cells. Some bacteria appeared to have a small pouch with leaked contents, which had traversed the damaged membrane (Figure 3C, D).

### Field tests

**Figure 4:**
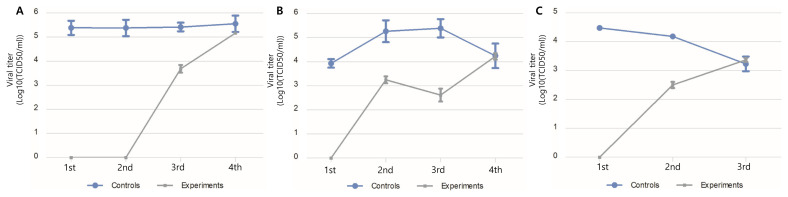
Efficacy of Si-QAC in a fully operational hatchery: all efficacy tests were performed once per washing step of the hatchery equipment (A, egg tray; B, chick-generating box) and space (C, egg-incubating room). Si-QAC, 3-(trimethoxysilyl)-propyldimethyloctadecyl ammonium chloride.

Field tests were conducted at a fully operational hatchery. The test surfaces were treated with Si-QAC once, and viral titers from egg trays (2-day interval), chick-hatching trays (3-day interval), and an egg incubation facility (18-day interval) were evaluated. Disinfection effects were observed in the egg trays and chicken-hatching trays until the third assessment. The disinfection effect was maintained in the incubation facility until the second assessment, which was 36 days after artificial contamination with NDV (Figure 4).

## DISCUSSION

In the poultry industry, pathogens such as avian influenza virus and *Salmonella* spp, are harmful to animal health, farmers economic situation, by extension, poultry workers and consumers health. There is much evidence of these pathogens being transmitted to farms because of workers’ activities and tools used. Inappropriate defective disinfection could introduce pathogens to the farms and spread them to animals. Our results provide a basis for using Si-QAC as a surface-coating disinfectant in farm environments. A previous report showed that Si-QAC has chemical disinfec-tion effects through its quaternary ammonium component and that its organosilicon component causes physical damage to the cells of pathogens [16]. Further, two mechanisms underlying the physical disinfection activity of Si-QAC have been suggested [17]. The first mechanism is the polymeric spacer effect, which is induced by long-chain cationic polymers that penetrate the outermost membrane of microbes [18]. The other is the phospholipid sponge effect, which induces ion exchange between short-chain polymers and microbial membranes, which results in the loss of membrane integrity [19].

Importantly, Si-QAC appears to be safe for use by humans; furthermore, it is a non-leaching material [8]. This material does not cause skin irritation [20]. In addition, Si-QAC showed no adverse effects in an inhalation toxicity test in rats over 90 days (data not shown). Moreover, it has a high safety level based on its 50% oral lethal dose, which is greater than 5,000 mg/kg in rats (data not shown). These data suggest that Si-QAC can be used safely with farm animals.

This study is the first report of Si-QAC application on farms. Although most results suggest that Si-QAC can be a potential disinfectant, additional data are required for maximizing the use of this disinfectant in different farm facilities. In livestock facilities, hot water high-pressure cleaning is routinely performed. This process may peel off the Si-QAC-coated layer and gradually decrease the disinfection efficacy. Information regarding factors such as the coating durability and optimal Si-QAC chain length for disinfecting surfaces to eliminate a diverse range of pathogens requires further elucidation.
